# Supplementary material for: Reverse redistribution-like change on dipyridamole-stress 99mTc-tetrofosmin imaging in a patient with angiographically mild coronary artery stenosis
Source: J Nucl Cardiol. 2021 Mar 9;28(3):1182–5. doi: 10.1007/s12350-021-02553-6 (PMC8249293; doi:10.1007/s12350-021-02553-6)

Reverse redistribution-like change on dipyridamole-stress ^99m^Tc-tetrofosmin imaging in a patient with angiographically mild coronary artery stenosis

**Short title: Reverse redistribution-like change on MPI**

Tadao Aikawa, MD, PhD;^1,2^ Naohiro Funayama, MD;^1^ Daisuke Sunaga, MD, PhD;^1^Keigo Kayanuma, MD;^1^ Noriko Oyama-Manabe, MD, PhD;^2^ and Daisuke Hotta, MD, PhD^1^

^1^Department of Cardiology, Hokkaido Cardiovascular Hospital, 1-30, Minami-27, Nishi-13, Chuo-ku, Sapporo, 064-8622, Japan

^2^Department of Radiology, Jichi Medical University Saitama Medical Center, 1-847 Amanuma-cho, Omiya-ku, Saitama 330-8503, Japan

**Address for Correspondence:**

Dr. Tadao Aikawa

Department of Cardiology

Hokkaido Cardiovascular Hospital

1-30, Minami-27, Nishi-13, Chuo-ku, Sapporo 064-8622, Japan

Tel.: +81-11-563-3911, Fax: +81-11-551-3109

Email: [tadao.aikawa@jichi.ac.jp](mailto:tadao.aikawa@jichi.ac.jp)

**Keywords:** ^99m^Tc-tetrofosmin, myocardial perfusion imaging, reverse redistribution

Word counts: 300

A 50-year-old man with a 1-month history of chest pain presented to our hospital. Transthoracic echocardiography showed normal left ventricular wall motion. Coronary computed tomography angiography revealed a mild stenosis with low-density non-calcified plaque in the proximal left anterior descending coronary artery (LAD) (**Figure 1A, yellow arrows**); therefore, the patient underwent a 1-day protocol of dipyridamole stress and rest ^99m^Tc-tetrofosmin myocardial perfusion imaging (MPI). Baseline electrocardiography was normal; however, he developed angina 8 minutes after the start of 0.56 mg/kg (0.14 mg/kg/min for 4 minutes) of intravenous dipyridamole infusion and his electrocardiogram showed ST-segment elevation in the precordial leads (**Figure 2**). After intravenous aminophylline with sublingual nitroglycerin was given, the ST-segment elevation was gradually resolved. Stress MPI showed no perfusion defect (**Figure 1B**) with abnormal wall motion in the anterior and septal walls on gated MPI (**Supplementary Material**). He had recurrent angina after the stress MPI. Rest MPI at 2 hours after the stress test showed reverse redistribution-like reduced uptake in the LAD territory (**Figure 1B)** with normal left ventricular wall motion on gated MPI (**Supplementary Material**). As with the coronary computed tomography, invasive coronary angiography via the right radial artery demonstrated the mild stenosis in the proximal LAD (**Figure 3, yellow arrow**). Intravascular ultrasound and optical coherence tomography images showed coronary plaque with neovascularization (**Figure 3, red arrows**) and small thrombi (**Figure 3, white arrows**) at the minimum lumen area site, indicating the increased vulnerability of the coronary plaque.^1^ Percutaneous coronary intervention (PCI) with a drug-eluting stent (4.0×33 mm) was successfully performed (**Figure 3, red arrows**). On the day following the PCI, he underwent cardiopulmonary exercise testing and did not present with chest pain at peak exercise (VO_2_ at peak was 22.5 mL/kg/min [6.4 METs]).

Intravenous dipyridamole and adenosine are widely used for pharmacological stress MPI to increase coronary blood flow. Previous studies reported that maximal coronary blood flow velocity is reached less quickly after the start of dipyridamole infusion than that of adenosine infusion (mean interval ± standard deviation, 287 ± 101 vs. 55 ± 34 s; p <0.0001),^2^ indicating that myocardial ischemia occurred after the tracer injection (7 minutes after the start of dipyridamole infusion) in this case. This case also suggested that dipyridamole-induced myocardial ischemia has a potentially harmful effect on vulnerable coronary plaque. Therefore, caution should be exercised when using dipyridamole for stress MPI.

**Acknowledgments**

We thank Atsushi Nagase for his technical assistance. This work was supported in part by JSPS KAKENHI (grant number 17K10349).

**Conflict of Interest**

None.

**Reference**

1. Kubo T, Imanishi T, Takarada S, Kuroi A, Ueno S, Yamano T et al. Assessment of culprit lesion morphology in acute myocardial infarction: ability of optical coherence tomography compared with intravascular ultrasound and coronary angioscopy. J Am Coll Cardiol 2007;50:933-9.

2. Rossen JD, Quillen JE, Lopez AG, Stenberg RG, Talman CL, Winniford MD. Comparison of coronary vasodilation with intravenous dipyridamole and adenosine. J Am Coll Cardiol 1991;18:485-91.

**Figure legends**

**Figure 1**. **A:** Coronary computed tomography angiography revealed a mild stenosis (*yellow arrows*) with low-density non-calcified plaque in the proximal left anterior descending coronary artery (LAD). **B:** Dipyridamole stress and rest ^99m^Tc-tetrofosmin myocardial perfusion imaging (MPI). Stress MPI (300 MBq of ^99m^Tc-tetrofosmin) showed no perfusion defect, whereas rest MPI (900 MBq of ^99m^Tc-tetrofosmin) 2 hours after the stress test showed reduced uptake (reverse redistribution) in the LAD territory.


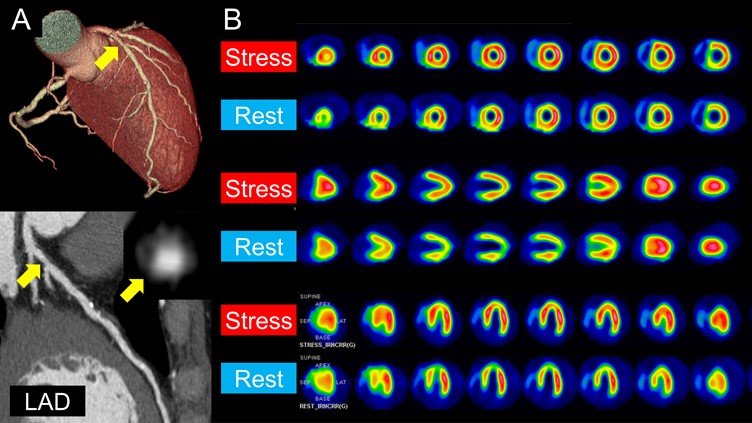


**Figure 2**. Serial electrocardiographic changes. Baseline electrocardiography before the dipyridamole infusion was normal. He developed angina 8 minutes after the start of 0.56 mg/kg (0.14 mg/kg/min for 4 minutes) of intravenous dipyridamole infusion. At 10 minutes after the start of dipyridamole infusion, his electrocardiogram showed ST-segment elevation in leads V1-5. CP = chest pain, CTCA = computed tomography coronary angiography, MPI = myocardial perfusion imaging, PCI = percutaneous coronary intervention.


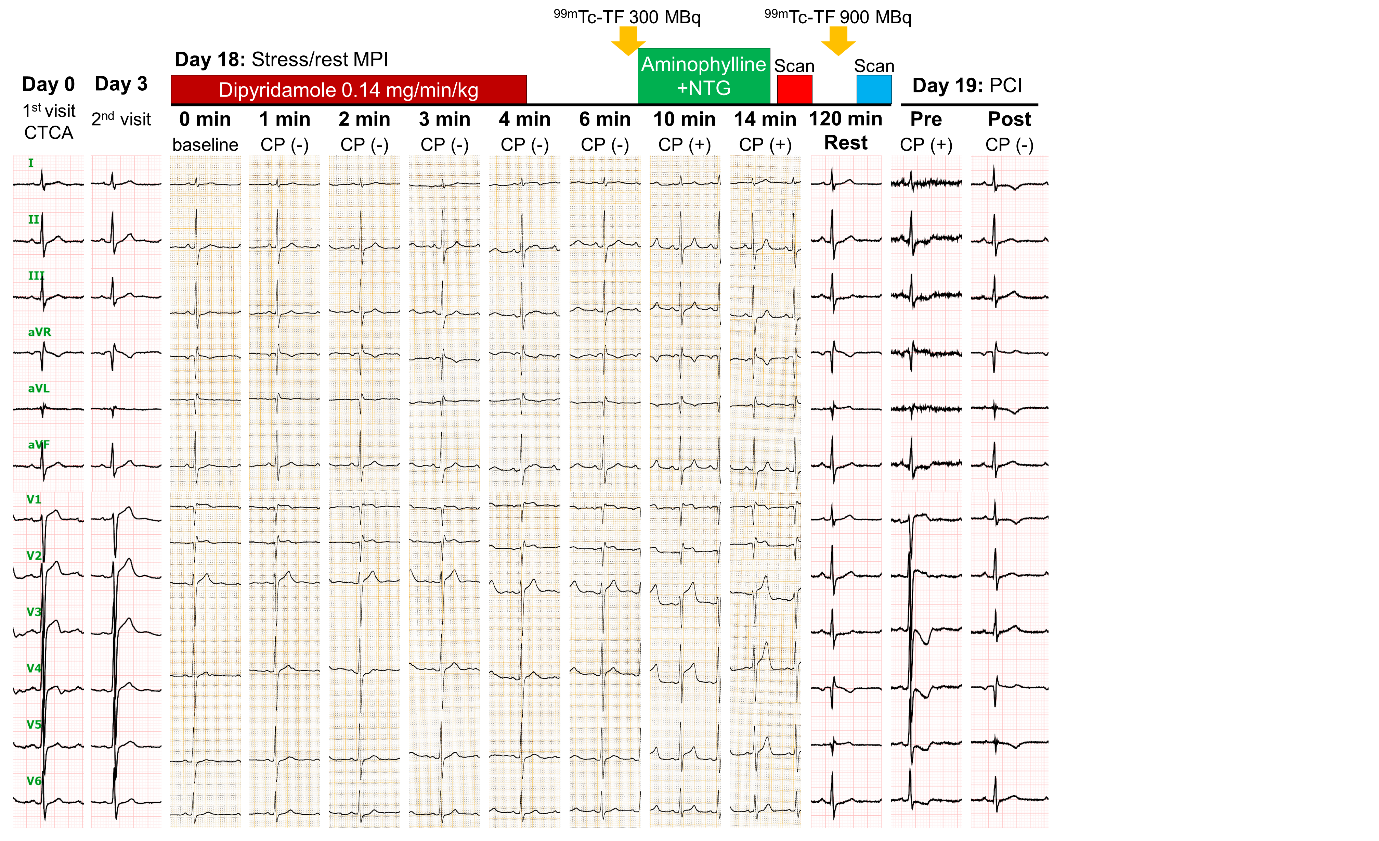


**Figure 3**. Invasive coronary angiography demonstrated a mild stenosis in the proximal left anterior descending coronary artery (*yellow arrow*). Intravascular ultrasound (IVUS) and optical coherence tomography (OCT) images showed coronary plaque with neovascularization (*red arrows*) and small thrombi (*white arrows*) at the minimum lumen area site, indicating the increased vulnerability of the coronary plaque. Percutaneous coronary intervention (PCI) with a drug-eluting stent (4.0 × 33 mm) was successfully performed (*blue arrow*).


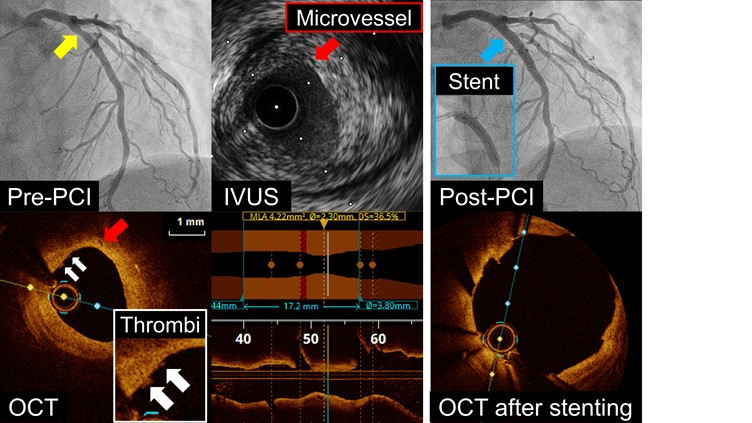

Supplement: Supplementary file 1 — Supplementary material 1 (DOCX 1944 kb) [file 12350_2021_2553_MOESM1_ESM.docx]
